# High-resolution two-photon polymerization: the most versatile technique for the fabrication of microneedle arrays

**DOI:** 10.1038/s41378-021-00298-3

**Published:** 2021-09-03

**Authors:** Zahra Faraji Rad, Philip D. Prewett, Graham J. Davies

**Affiliations:** 1 School of Mechanical and Electrical Engineering, University of Southern Queensland, Springfield Central, QLD 4300 Australia; 2 Department of Mechanical Engineering, University of Birmingham, Birmingham, B15 2TT UK; 3 Oxacus Ltd, Dorchester-on-Thames, OX10 7HN UK; 4 Faculty of Engineering, UNSW Australia, Kensington, NSW 2052 Australia; 5 College of Engineering and Physical Sciences, School of Engineering, University of Birmingham, Birmingham, B15 2TT UK

**Keywords:** Nanofabrication and nanopatterning, NEMS

## Abstract

Microneedle patches have received much interest in the last two decades as drug/vaccine delivery or fluid sampling systems for diagnostic and monitoring purposes. Microneedles are manufactured using a variety of additive and subtractive micromanufacturing techniques. In the last decade, much attention has been paid to using additive manufacturing techniques in both research and industry, such as 3D printing, fused deposition modeling, inkjet printing, and two-photon polymerization (2PP), with 2PP being the most flexible method for the fabrication of microneedle arrays. 2PP is one of the most versatile and precise additive manufacturing processes, which enables the fabrication of arbitrary three-dimensional (3D) prototypes directly from computer-aided-design (CAD) models with a resolution down to 100 nm. Due to its unprecedented flexibility and high spatial resolution, the use of this technology has been widespread for the fabrication of bio-microdevices and bio-nanodevices such as microneedles and microfluidic devices. This is a pioneering transformative technology that facilitates the fabrication of complex miniaturized structures that cannot be fabricated with established multistep manufacturing methods such as injection molding, photolithography, and etching. Thus, microstructures are designed according to structural and fluid dynamics considerations rather than the manufacturing constraints imposed by methods such as machining or etching processes. This article presents the fundamentals of 2PP and the recent development of microneedle array fabrication through 2PP as a precise and unique method for the manufacture of microstructures, which may overcome the shortcomings of conventional manufacturing processes.

## Introduction

**Fig. 1:**
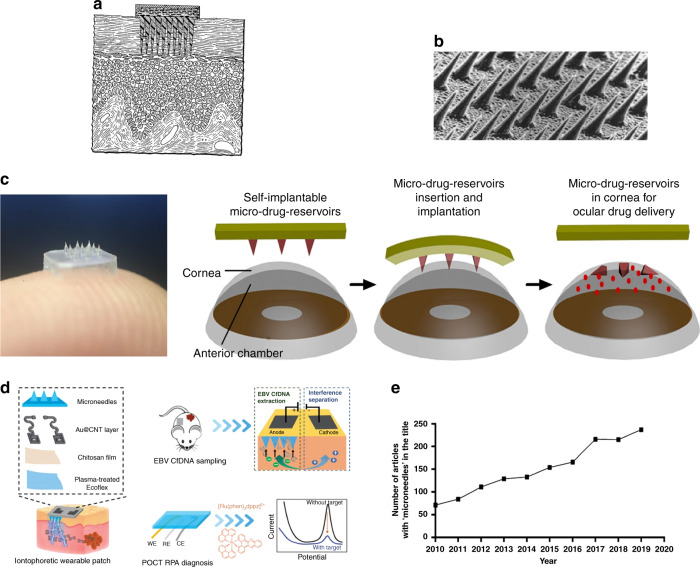
A brief overview of microneedle technology initiation and development. **a** The concept of microneedles was initially developed in 1976^3^. Reproduced with permission from ref. ^3^. Copyright 1976, US Patent US3964482A. **b** The early focus in the 1990s was on transdermal drug delivery, with the first such microneedle device fabricated using the DRIE process^4^. Reproduced with permission from ref. ^4^. Copyright © Elsevier. **c** Recent developments in the technology for controlled ocular drug delivery using a flexible polymeric and biodegradable microneedle patch, representing a nontransdermal application of microneedles^97^. Reproduced with permission from ref. ^97^. Copyright © 2018, Nature Publishing Group. **d** Schematic illustration of an iontophoretic wearable microneedle device and electrochemical microfluidic platform for the extraction of EBV CfDNA from the ISF of mice^98^. Reproduced with permission from ref. ^98^. Copyright © 2020, Wiley-VCH Verlag GmbH & Co. KGaA, Weinheim. **e** Increase in the number of journal articles published covering microneedle applications since 2010, indicating the importance of the technology^99^. Reproduced with permission from ref. ^99^. Copyright © Springer

Microneedle arrays are micrometer-sized structures designed to reduce the risk and difficulty in the administration of hypodermic needle-based injections. Some of these difficulties include the need for medically trained staff for administration, needle phobia, and needle injuries. Microneedles are minimally invasive and have shown capabilities to sample biofluids and deliver a variety of nanoparticles and molecules to the human body for drug and vaccination applications^1,2^. The initial microneedle idea was proposed in 1976^3^, but due to the limitations of manufacturing techniques, the fabrication of the first microneedle prototypes occurred only in the 1990s, when advancements in micromanufacturing enabled the creation of microstructures^4^ (Fig. 1). Interest in microneedle-based medical devices is growing rapidly as healthcare systems recognize the importance of small, portable medical devices for point-of-care diagnostics and the effective and rapid administration of drugs and vaccines (Fig. 1e). To be able to penetrate the skin, microneedles should have specific physical properties and precise geometries. In this regard, penetration and mechanical properties are important aspects that need to be addressed, to determine whether microneedle arrays can pierce the skin without breaking. A thorough understanding of the skin structure, anatomy, and cellular and outer surface characteristics as a living unit is crucial for the successful design and fabrication of microneedles. The importance can be better realized when considering that microneedle arrays should bypass the skin layers to access the desired section of the skin. Due to the elastic nature of the skin, the microneedle insertion depth strongly depends on the amount of deformation that occurs around the insertion site on the skin^5^. To bypass this problem, either the insertion force may be increased or the microneedle sharpness can be increased. As increasing the applied force can cause discomfort to the patient and may result in microneedle breakage, it is more effective to increase the tip sharpness for easier penetration of the microneedle into the skin^6^. The study conducted by Davis et al.^7^ showed that there is a linear relationship between the microneedle insertion force and the microneedle tip interfacial area, as the microneedle tip reduces the force of fracture. A force range of 0.1–3 N was reported to be required for microneedle insertion into the skin depending on the area of the tip. The tip size usually depends on the manufacturing technique and the material used. The tip diameter can be as small as 500 nm depending on the manufacturing accuracy. Another study confirmed that to overcome the high hydrostatic pressures induced on skin during microneedle penetration^8^, it is essential to fabricate sharp microneedles. A needle tip will penetrate the human epidermis if it applies tensile stress at the point of contact beyond the ultimate strength of skin (27.2 ± 9.3 MPa). The ultimate strength of skin varies with age and body location^9^. The sharper the needle tip is, the more concentrated the tensile force at the point of contact. The tip also needs to be harder than the skin for it to penetrate. Considering the above factors, the successful application of microneedle arrays greatly depends on the tip sharpness and robust structure of the microneedle arrays. This requires access to manufacturing capabilities that allow high structural strength, flexibility, and resolution.

In the past decade, researchers have used a variety of manufacturing techniques, including lithography, dry and wet etching, drawing lithography, micromolding, and laser cutting, to produce microneedle arrays in different geometrical patterns from materials such as silicon, glass, ceramics, metals, and synthetic and natural polymers, including biodegradable polymers such as carbohydrates. Each class of material has its own advantages and disadvantages, and requires specific manufacturing tools for production^10^. Among all the materials used to manufacture microneedles, polymers have the greatest potential for mass manufacturing due to their combination of mechanical properties, biocompatibility, degradability, and ease of replication. Polymeric microneedles are mainly fabricated via hot and soft embossing, drawing lithography, three-dimensional (3D) printing, 2PP, casting, and laser micromachining. A variety of molding techniques can be utilized to replicate polymeric microneedle arrays; usually, a negative mold made from polydimethylsiloxane (PDMS) is used to create microneedle replicas. Manufacturing technicities such as etching, lithography, and 2PP may be used to produce master microneedle templates that are typically made from materials such metal, silicon, and polymer.

In recent years, technologies such as 3D printing and 2PP have found their way into both industry and academia. These technologies are examples of additive manufacturing but are best described as additive, layer-by-layer processes to create 3D structures that use a model generated by computer-aided-design (CAD) tools to fabricate a 3D object. Among all 3D manufacturing techniques, high-precision 2PP has produced sharper microneedle arrays with more versatile designs. In addition, unlike other microneedle microfabrication methods, rapid prototyping technologies do not require clean room facilities with high capital and running costs, and there is no need for harsh processing environments such as hydrofluoric acid (HF), deep ultraviolet exposure, or reactive-ion etching. In addition, components with complex geometries can be fabricated in a shorter time and with less technical expertise. This is very advantageous for the fabrication of microneedle patch arrays, in particular the integration of other microfluidic components that may be required for point-of-care diagnostics or drug delivery. In the 2PP nanolithography technique, a femtosecond or picosecond laser is used. The polymerization is started by two-photon absorption (TPA) triggered by a focused laser pulse, which provides a nonlinear energy distribution centered at the laser’s focal point applied to the photosensitive materials^11^. The photoinitiator (PI) molecules in the photosensitive resins start the polymerization process upon absorption of this energy at regions known as “polymerization voxels,” where the absorption energy exceeds a specific threshold of the resin, thereby forming the polymerized 3D micro/nanostructure. Recent commercialization of the 2PP microtechnology by companies such as Nanoscribe GmbH of Karlsruhe, Germany, with their Photonic Professional GT system, has enabled the precise manufacture of devices at submicrometer resolution. This technology enables reproducible production of complex structures in a short manufacturing time and with exceptional flexibility, using femtosecond laser pulses from a near-infrared (NIR) laser beam via a controlled printing head to selectively polymerize an uncured photosensitive resin^12^.

2PP has been used by researchers to manufacture a wide range of microneedle arrays, geometries, and materials, including modified ceramics^13^, acrylate-based polymers^14,15^, inorganic–organic hybrid polymers^16,17^, water-soluble materials^18^, and polyethylene glycol^19^. In addition to microneedle manufacturing using 2PP, this technology has been used for the fabrication of micromechanical systems, including biomedical devices^20^, plasmonic components^21^, scaffolds for tissue engineering^22–24^, and micro-optical components^25–27^.

**Fig. 2:**
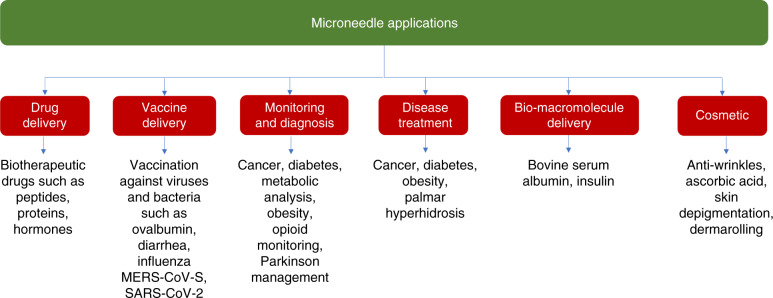
The most common applications of microneedle-based devices. To our knowledge, all of the applications, except the cosmetic applications (e.g., Dermaroller^®^), are in the experimental phase

Microneedle arrays have been introduced primarily for drug delivery and point-of-care diagnostics to improve the quality of healthcare delivery systems. These devices have been used for a wide range of applications, including drug and vaccine delivery, the sampling of biofluids for monitoring and diagnostic purposes, and cosmetic applications. Figure 2 shows the most common applications of microneedle devices to date. Microneedles have proved to be pain free by passing the stratum corneum (SC) of the skin and penetrating the viable epidermis without stimulating the nerve fibers. Microneedles may be integrated into biosensors, micropumps, microfluidic chips, and microelectronic devices for different applications.

Most of the existing review papers on 2PP consider a broad range of biomedical applications. To our knowledge, the only prior work on reviewing 2PP technology for the fabrication of microneedles was published in 2010^19^. This paper provides an introduction to 2PP technology, with emphasis on utilizing this method for the fabrication of microneedle structures for biomedical applications. The skin structure and its interaction with microneedle arrays are briefly reviewed. As discussed earlier, the long-standing challenge for microneedle-based devices is to fabricate structures with great accuracy, precision, and strength that can withstand a range of applied forces during penetration into the skin. This demands a manufacturing technique that enables structures to be freely designed without the manufacturing constraints of conventional manufacturing methods. In this regard, 2PP has demonstrated mechanically strong structures, reproducible outcomes, and relatively simple manufacturing processes, enabling the fabrication of complete devices in a single step and eliminating the need for assembly to integrate multiple parts.

## Skin structure and anatomy

The skin is a complicated living substance that is composed of heterogeneous layers. It protects the body from unwanted and detrimental environmental objects or effects. Human skin is made of three layers as follows: epidermis, dermis, and hypodermis. The SC is the outermost layer of the epidermis; it is a flat dead tissue packed with keratin fibers (corneocytes) with a thickness of 15–20 µm located in lipid areas^28,29^. Overcoming the SC barrier and creating a pathway through it are the key problems when designing microneedles for transdermal drug delivery and fluid sampling^30^. The epidermis is an upper avascular cellular layer that is connected to the substance-rich dermis and collagen, and the underlying subcutaneous fat tissue^31^. Nerve endings and living cells are located in the viable epidermis just underneath the SC; this epidermis is 50–150 µm in thickness and has no blood vessels. The bulk volume of the skin is composed of dermis, which is below the epidermis^29^; elastin and collagen fibers are the main components of the dermis. Capillaries, living cells, sweat glands, hair follicles, and nerves can all be found within the dermis, which has an approximate thickness of 2000 µm. Capillaries are located in the dermis ~500–2000 µm below the surface of the skin. The epidermis and the dermal layers hold interstitial fluid (ISF) as well as the structural membranes and fibers. The ISF is a water-based medium that surrounds the cells and is responsible for transferring ions and nutrients to and from them^29,32^. Skin properties vary according to different factors, such as age, hydration level, and body zone; thus, the thicknesses of the skin layers provided are approximate.

## Two-photon fabrication of microneedles

Three-dimensional laser lithography based on 2PP is a leading technology in ultraprecise 3D micro- and nanofabrication for a variety of applications in addition to microneedles, including micro-optics, photonics, and microfluidics^33^. It has several advantages over other conventional microfabrication techniques such as deep reactive-ion etching (DRIE), laser ablation, microstereolithography (µSL), drawing lithography, droplet-born air blowing, and chemical isotropic etching, including the following: (i) the nonlinear response of the photoresists produces superior resolution (approximately tens of nanometers), (ii) the method creates complex 3D structures directly from a CAD drawing, and (iii) it allows fabrication of tall microstructures such as high-aspect -ratio (e.g., 5 : 1) microneedles.

Conventional 3D printing techniques, including fused deposition modeling and stereolithography (SLA) or µSL, are used^34–38^ to produce microneedle arrays, but their resolutions are several orders of magnitude less than that of 2PP, and they are incapable of directly forming an object with a controlled feature size <1 µm^39^. The SLA technique uses UV light to cure a photosensitive material using a UV laser scan, after which a fresh photoresist layer is added. The writing process is continued layer-by-layer until completion of the structure. Economidou et al.^38^ used SLA to fabricate solid microneedles and coated drugs (e.g., insulin) on the surfaces of microneedles by inkjet printing. Lu et al.^37^ utilized the µSL method to create arrays of drug-loaded microneedles with a 700 µm-long base and a 300 µm-long conical tip from poly(propylene fumarate). Commercially available SLA systems do not have sufficiently high resolution to produce features in the size range that is required for most microneedle applications.

The choice of manufacturing techniques for producing microneedles is dependent on the material properties, fabrication cost, and desired length and shape of the microstructure. Table 1 provides a summary of the main advantages and disadvantages of the most common methods used to manufacture microneedles.

**Table 1:** Summary of the main advantages and disadvantages of the most common fabrication methods used to manufacture microneedles

Fabrication methods	Advantages	Disadvantages	Ref.
Plasma etching	• Equipment more widely available • Multiple arrays can be manufactured at the same time	• Little control over geometric features when fabricating longer structures (>~500 μm in height) • Fabrication of geometrically complex microstructures is not possible • Design geometry and fabrication method are restricted to structures that are normal to the wafer surface • Multiple fabrication steps are required • The recipes developed by one etch system are not readily applicable to another etch system • Undercutting can occur when etching high-aspect-ratio walls • Sidewall roughness of the etched profiles may require further wet etching to smooth • High fabrication cost • Low control of microneedle tip angles • Requires a clean room facility	^67,83,84^
Chemical wet etching	• More widely available equipment • High selectivity • More controllable etching rate • Low cost	• Undercutting • Limited to low-aspect-ratio fabrication • Poor reproducibility • Requires a clean room facility • Si wet etch rate depends on crystal orientation, with KOH used to give faceted shapes	^67,83,85^
Photolithography	• Low cost	• Limited materials, e.g., SU-8 epoxy resin • Multistep process • Poor reproducibility • Time consuming • Requires a clean room facility	
2PP	• High feature resolution • Versatility • Easy to control • High reproducibility • High accuracy and control	• High equipment cost • Slow process for arrays (typically > 3 h). This is usually used for the production of the master for replication. The micromolding is then simple and rapid.	^12,67,73^
Micromolding	• High reproducibility and precision • Reusability of the molds • Cost-effectiveness	• Requires other fabrication techniques to create the mold, e.g., 2PP, laser micromachining	^10,18^
Laser cutting and laser ablation	• High-aspect-ratio fabrication	• Time consuming • High cost • Scale-up challenges • Low resolution	^86^
Drawing lithography	• High-aspect-ratio fabrication	• Scale-up challenges • Limited control • Requires high temperature; not suitable for heat-sensitive drugs	^87–89^
Microstereolithography (μSL)–3D printing	• Low cost • Flexibility to create complex structures • Rapid prototyping	• Low resolution • Limited materials	^34,38,90^
Droplet-born air blowing	• Low cost • No heat or UV irradiation is required • Fast	• Limited to dissolving microneedles • Limited control • Poor design flexibility	^89,91,92^

## Two-photon polymerization fundamentals

### Two-photon absorption

**Fig. 3:**
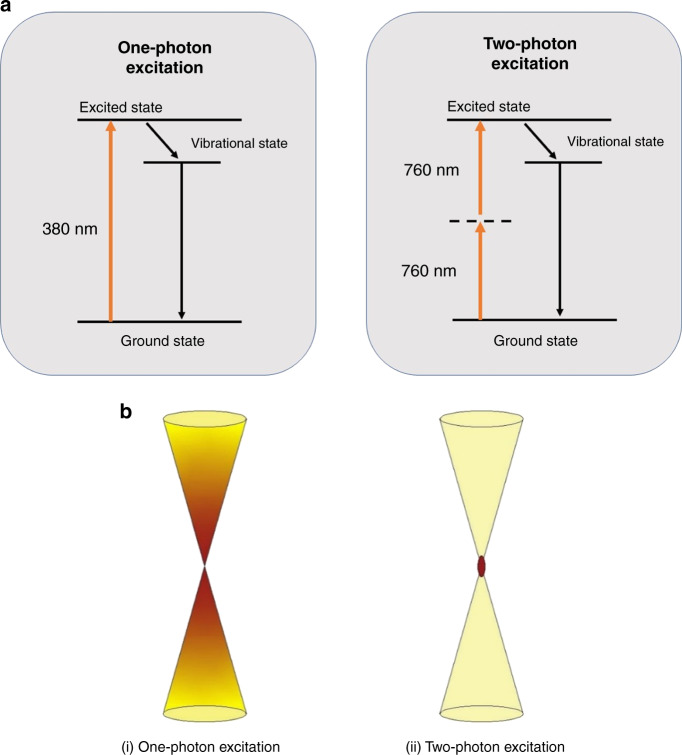
In OPA excitation occurs along the trace of the beam whereas in TPA the excitation is limited to the voxel around the focal point. **a** Comparison of the absorption energy of a single photon by UV light and two photons by near-infrared light. **b** Schematic picture comparing the excitation volume of one-photon excitation (i) and two-photon excitation (ii). In 2PP, regions outside the laser focus are less likely to exceed the polymerization threshold of the photoresist. This phenomenon allows the fabrication of complex 3D structures, as the proximity effect in TPA is significantly less than that in OPA.

Two-photon polymerization, also known as direct laser writing (DLW), femtosecond laser writing, dip-in laser lithography (DiLL), multiphoton SLA, or 3D laser lithography^40^, is a nonlinear optical process based on TPA theory. The concept of TPA was first described by Göppert-Mayer in 1931^41^. However, due to the necessity of applying high photon intensities, the theory was not experimentally tested until the development of ultrafast lasers. Thirty years later, Kaiser and Garrett^42^ first demonstrated the TPA phenomena in 1961. In TPA, a molecule is excited from its ground state to a higher energy level by the simultaneous absorption of two photons with different or equal frequencies. Figure 3 shows a comparison of the absorption energy of a single photon by UV light and two photons by NIR light. Figure 3a shows the Jablonski diagrams of the one-photon and TPA processes, where in the case of TPA, two photons of equal energies are excited. The total energy of the two photons is equal to the difference between the upper and lower energy states of the molecule, where each photon, with the same frequency, has half the energy required for single-photon excitation^43^. One-photon absorption (OPA) is a linear mechanism in which the energy absorbed is a linear function of the light intensity *I*, whereas TPA is nonlinear with the absorption ∝*I*^2^. Thus, for a focused laser providing a round Gaussian beam, the fall-off in absorbed intensity with the distance from the center of the spot, in the case of TPA, has twice the exponential decrement of OPA, which provides a significant increase in resolution on absorption by the photoactive polymer. The resist exposure mechanism shown in Fig. 3a involves decay from the photon excited intermediate state to a vibrational state, which then causes scission of polymer bonds in the case of a positive-tone resist or the formation of crosslinking bonds for a negative-tone resist.

2PP is different from conventional SLA methods, where scanning the surface of a photosensitive material by using a UV laser creates two-dimensional patterns of polymerized material by single-photon absorption and the fabrication of 3D microstructures is only possible layer-by-layer. In 2PP, the fabrication of 3D objects is not limited to the layer-by-layer method and microstructures are created by DLW of a transparent photosensitive material that is highly absorptive in the UV range and normally transparent in the infrared region. Thus, arbitrary 3D microstructures can be fabricated by high-intensity ultrashort femtosecond NIR laser pulses (up to 10^12^ W/cm^2^/sr) when the simultaneous energy of two photons exceeds the absorption energy threshold of the photosensitive material^44^. Consequently, free radicals are released by PI molecules^45^ and 3D microstructures are created via photochemical reactions induced by TPA, by nonlinear photon absorption. In this technique, a high-energy femtosecond NIR (~800 nm) laser is used as the optical source to selectively polymerize a photosensitive material by emitting light onto uncured resin, to polymerize the material in a highly localized region. Photosensitive materials are usually composed of transparent photosensitive polymeric monomers with functional groups at the NIR wavelength and PIs with absorption near the two-photon excitation wavelength^46^. In commercial systems, the laser pulse photon intensity is significantly high (4 × 10^12^ W/cm^2^/sr). However, in comparison to a conventional laser with ~1–10^3^ Hz, the generated laser power is much lower, as the repetition period of ultrafast pulses is extremely shorter. Thus, femtosecond NIR (~800 nm wavelength) Ti:sapphire lasers with high peak power and short pulse width are commonly used in the 2PP process^44^.

### Polymerization process

The nonlinear property of the optical process allows the laser to tightly focus onto a spot and creates the smallest building block of the 3D construction known as the volume pixel (voxel), from which the nano/micro 3D structure will be fabricated^45^. From this voxel-by-voxel process, precise microstructures will be created by a tightly focused laser beam without using a photomask. In OPA, excitation occurs along the trace of the beam, whereas in TPA the excitation is limited to the voxel around the focal point (Fig. 3b). The voxel has an ellipsoidal shape and different parameters including the objective numerical aperture (NA), laser mode, and refractive index difference between the immersion system and the resist determine the size and shape of the voxel and the corresponding laser focus intensity distribution^45,47^. The photosensitive materials used for 2PP are resins with an acrylic base, which are composed of a PI and a combination of monomers and oligomers. The polymerization process starts due to the presence of PI molecules, which generate active species (radicals) by TPA in a highly localized region around the center of the focused beam, leading to the formation of a solid voxel for the fabrication process^48,49^. A wide range of readily available and low-cost photosensitive resins have been used for 2PP, including inorganic–organic hybrid materials (Ormocers)^48^, urethane acrylate monomers^50^, acrylic-based prepolymers^51^, single-walled carbon nanotube-dispersed resins^52^, gelatin hydrogels^53^, zirconium sol-gels^54^, and water-soluble materials^18^.

The resolution of 2PP is easily adjustable by changing the voxel dimensions and the objective lens. The voxel sizes and time of fabrication for a structure have an inverse relationship; thus, the fabrication efficiency and processing costs may be optimized by selecting an appropriate objective^48^. The rate of photosensitive material polymerization is proportional to the square of the laser intensity in the system. Therefore, by using a high-NA objective lens equipped with a femtosecond and/or picosecond laser pulse, high-resolution (<100 nm) structures can be achieved^55^.

A typical photopolymerization process occurs through the following steps: (1) initiation, (2) propagation, and (3) termination^56^. To begin the initiation process, an active PI needs to be used, to allow chemical polymerization to occur and generate radicals. To maximize the potential of the 2PP process and to achieve a desired initiation rate, it is important to select highly photochemically active PIs in the initiation phase. The properties of the final structure, such as its viscosity and hardness, as well as the chemical polymerization mechanism and the rate of polymerization reaction depend critically on the PIs^57^. A wide range of commercial PIs are available for biomedical applications, such as Rose Bengal^58^, 6-trimethylbenzoylphosphinate^59^, lithium phenyl-2,4^60^, methylene blue^61^, and Irgacure 369^62^. In the propagation step, the radicals generated in the initiation phase serve as the activator for the oligomers or monomers, resulting in the creation of monomer radicals that expand in a chain reaction. Finally, in the termination step, the two radicals join together^44,63^.

### Dip-in laser lithography

**Fig. 4:**
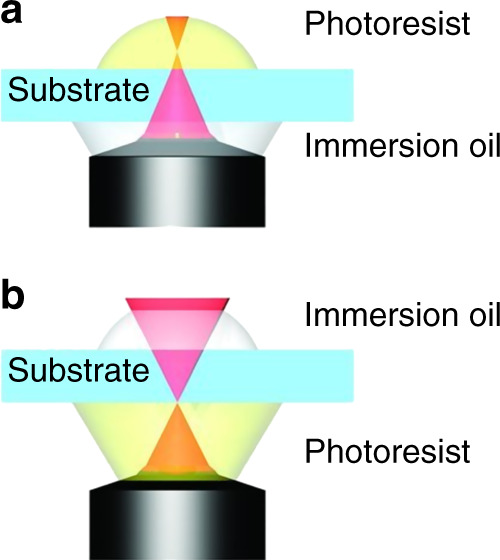
Conventional 3D direct laser writing lithography vs. dip-in 3D laser lithography. In **a** the fabrication of high structures is limited by the working distance of the microscope, whereas in **b** the achievable sample height is not limited by the working distance of the microscope. Reproduced with permission from ref. ^63^ Copyright © 2012 WILEY‐VCH.

In conventional DLW, photoresists are usually not refractive index-matched to the oil-immersion microscopic system, and refractive or reflective errors resulting from this mismatch lead to intense loss of laser power and resolution as the writing depth is increased. Furthermore, the height of the substrate is limited to the oil-objective working distance. In this regard, Nanoscribe GmbH invented a new laser lithography process called DiLL to overcome the drawbacks associated with conventional DLW. In this process, the objective lens is directly dipped into the liquid and an uncured photoresist acts as both a photosensitive and immersion medium in an inverted fabrication manner. The refractive index of the photoresist defines the focal intensity distribution. In DiLL processing, the objective working distance does not limit the height of the sample; therefore, structures with millimeter heights can be fabricated. Opaque substrates such as silicon can also be used in DiLL; however, transmissive substrates show better reflective illumination than opaque substrates^64^. Figure 4 illustrates the difference between the conventional DLW and DiLL systems.

### Photoresists

Most of the photosensitive materials commonly used in UV lithography can still be used for 2PP. The difference lies in the absorption of photons in both methods, which determines the spatial resolution of the 3D structure. The materials are typically in the form of gels, viscous liquids, or amorphous solids. Epoxy-based and acrylate-based resins are the most commonly used materials developed for single-photon absorption, normally polymerized by a Hg lamp at 365 nm or an excimer laser at 248 and 193 nm, and can still be used in 2PP^15,46,65^. Many photoresists are compatible with 2PP, although certain requirements need to be fulfilled, in particular the compatibility; the material (1) must be optically transparent to the laser wavelength^66^ and (2) needs to be a UV-sensitive photoresist capable of being polymerized by TPA, such as positive- and negative-tone photoresists. A wide range of optically transparent photosensitive materials has been commercially developed for the fabrication of miniaturized structures specifically for 2PP. In positive-tone photoresists, the material is initially solidified by heat or a UV laser. Subsequently, exposure to the NIR laser beam leads to the breakup of the photoresist polymer chains via photoacid degradation, creating smaller units that can be dissolved and removed in the development stage. Some examples of positive-tone photoresists are AZ® MIR 701, AZ® 5214, AZ® 9260, and AZ® 40XT^67^. In negative-tone photoresists, laser exposure crosslinks the polymer chains, which leads to the formation of the cured 3D object on a substrate, and finally, the unpolymerized resist is removed by a solvent developer. Some examples of negative-tone photoresists are acrylate-based photoresists, e.g., IP-series resists (Nanoscribe GmbH)^12^ and hybrid sol-gel Ormocer^®^ (Microresist Technologies)^68^, and epoxy-based photoresists, notably SU-8 (MicroChem)^69^. This type of photosensitive material is ideal for the fabrication of high-resolution microstructures. Some advantages of negative-tone and acrylic-based photoresists include low shrinkage, low stress, good adhesion to substrates, excellent chemical stability, a low proximity effect allowing the dense packing of submicrometer features, and easy handling effects such as photoresist drop casting^67,70^.

Nanoscribe GmbH has developed a selection of commercially available 2PP-compatible proprietary IP photoresists for 3D micro- and nanofabrication with high mechanical stability, low shrinkage, easy handling, and superior resolution. IP photoresists are negative tone and especially developed for DLW through the nonlinear absorption of femtosecond NIR laser beams in 2PP. IP-Dip, IP-S, IP-L780, and IP-Q resins are most suitable for the DiLL process, whereas IP-G 780 resin is preferred for the oil-immersion method. IP-S resin has shown great surface finish and smoothness, and high spatial resolution for the fabrication of microneedle arrays^12^. IP-Visio resin was recently developed by Nanoscribe GmbH with noncytotoxicity and low fluorescence properties for biomedical applications such as tissue engineering^46,71^. Among all IP photoresists, IP-S has been most frequently used for the fabrication of microneedle arrays in recent years (Table 2).

**Table 2:** A detailed list of microneedle arrays fabricated by 2PP.

MN (microneedle) structures	Fabrication	Bioapplication	Materials	Device/femtosecond laser pulse source	Ref.
Up to 1300 µm high	Master MN arrays fabricated by 2PP. Silicone MN array molds were fabricated from these templates and used to produce dissolving and hydrogel-forming MN arrays	Evaluated for their insertion in skin models and their ability to deliver model drugs to viable layers of the skin	IP-S resist	Nanoscribe GmbH (780 nm, a pulse width of 150 fs, a frequency of 40–100 MHz, and a maximum output of 45 mW)	^73^
210 µm High pyramids	Master MN arrays fabricated by 2PP. Silicone MN array molds were fabricated from these templates and used to create an epoxy replica	Penetration efficiency of MN arrays (3 × 3) was tested ex vivo on human skin and on a male volunteer	OrmoComp^®^ and 1 wt% of Ciba® IRGACURE^®^ 2959 (Sigma-Aldrich)	Workshop of Photonics	^16^
Pyramidal heads with undercut stems, 750 µm in height, 150 µm square base	Master MN arrays fabricated by 2PP. Silicone MN array molds were fabricated from these templates and used to create dissolvable MNs	Vaccination with antigen-loaded MN arrays	IP-S resist	Nanoscribe	^18^
430 µm MN height; total height (including the base) is 585 µm	MN-negative mold fabricated by 2PP. Electrodeposition method was used to fill the mold with copper and create the final MN	Drug delivery into the inner ear	IP-S resist	Nanoscribe	^77^
Not mentioned	MN directly fabricated through 2PP	Drug delivery purposes	IP-S resist	Nanoscribe	^20^
250 and 300 µm Cone-shaped and pyramid-shaped structures	MN directly fabricated through 2PP	Point-of-care diagnostics	OrmoComp® (microresist technology, Berlin, Germany), an organic/inorganic hybrid polymer	Ytterbium-doped femtosecond oscillator (Amplitude Systems, Mikan) with a pulse duration of 300 fs and a repetition rate of 55 MHz at 515 nm	^17^
Single MN 150 µm in height, 100 µm in diameter, and 500 nm in tip radius of curvature	MN directly fabricated through 2PP	Designed to pierce the guinea pig RWM for drug delivery purposes	IP-S resist	Nanoscribe	^78^
Height of 350 µm; diameter of 100 and 150 µm	MN directly fabricated through 2PP	Designed to pierce the human RWM for drug delivery purposes	IP-S resist	Nanoscribe	^80^
MNs mimicking mosquitoes	MN directly fabricated through 2PP	Designed for blood collection	IP-S resist	Nanoscribe	^93^
Height of 200 µm, tip radius of 500 nm, shank radius of 50 µm		For in vitro perforation; designed to pierce the guinea pig RWM for drug delivery purposes	IP-S resist	Nanoscribe	^79^
Hollow-bore pyramidal MNs with 1 mm height and 500 µm width	MN directly fabricated through 2PP integrated with electrode arrays and microfluidic channel, which are fabricated via photolithography, etching, and laser cutting processes	A lab-on-chip device for detecting proteins, including troponin and myoglobin	E-Shell 300 polymer	A Ti:Sapphire laser was used for 2PP at 800 nm, 150 fs, and 76 MHz	^94^
Hollow MNs with a 1450 µm height, 440 µm width, and 165 µm triangular bore	MN directly fabricated through 2PP integrated with microfluidic chip	Point-of-care diagnostics MN device for detecting potassium	E-Shell 300 polymer	A Ti:Sapphire laser was used for 2PP at 800 nm, 150 fs, and 76 MHz	^95^
Variable sizes of solid and hollow MNs with heights ranging from 375 to 750 µm and base diameters ranging from 125 to 250 µm	MN directly fabricated through 2PP	MN arrays used to inject quantum dots into porcine skin	E-Shell 300 (acrylate-based polymer)	Ti:Sapphire laser (60 fs, 320 mW, 780 nm)	^15^
Various sizes of MNs ranging from 750 to 500 µm and base diameters of 250 and 300 µm	Master MN arrays fabricated by 2PP. Silicone elastomer MN array molds were fabricated from these templates and were used to produce the final MN array from the E-Shell 200 polymer	Mechanical strength of different microneedle geometries examined for drug delivery	SR 259 (polyethylene glycol dimethacrylate)	Ti:Sapphire laser (60 fs, 94 MHz, 450 mW, 780 nm)	^96^
Height of 550 µm and base diameter of 150 µm	MN directly fabricated through 2PP	Delivery of a quantum dot solution to porcine skin	Ormocer^®^	Ti:Sapphire laser (60 fs, 94 MHz, 450 mW, 780 nm)	^68^
Solid MNs with 500 µm height and 200 µm base diameter	Master MN arrays fabricated by 2PP. Silicone elastomer MN array molds were fabricated from these templates and were used to produce the final MN array from Ormocer^®^	Drug delivery, providing antimicrobial functionality to MN arrays	SR 259 (polyethylene glycol dimethacrylate)	Ti:Sapphire laser (60 fs, 300 mW, 780 nm)	^76^
Solid MNs with 500 µm height and 150 µm base diameter	Master MN arrays fabricated by 2PP. Silicone elastomer MN array molds were fabricated from these templates and were used to produce the final MN array from the E-Shell 200 polymer	Drug delivery	SR 259 polymer (polyethylene glycol (200) diacrylate)	Ti:Sapphire laser (60 fs, 94 MHz, <450 mW, 780 nm)	^14^
800 μm Height, 150–300 μm base diameter	MN directly fabricated through 2PP	Drug delivery-MN penetrated cadaveric porcine adipose tissue	Ormocer^®^ US-S4	Ti:Sapphire laser (60 fs, 94 MHz, <450 mW, 780 nm)	^75^
750 μm Height and 200 μm base diameter	MN directly fabricated through 2PP	Drug delivery	Ormocer^®^	Ti:Sapphire laser (94 MHz, <450 mW, 780 nm)	^13^

### Design and pattern generation

In 2PP, the desired microstructure is initially created by CAD software. The 3D design model from the CAD program is then converted into an STL (Standard Tessellation Language) file. The STL file is then imported into another software package for slicing into layers and parameter setup to generate the interpreted general writing language (GWL) code for printing. Consequently, the GWL code is sent to the system equipped with a femtosecond laser source to manufacture the respective layers from the base layer by tightly focused laser beams exposing the photosensitive resins. Commercial 2PP systems typically utilize three scanning modes for photosensitive material polymerization as follows: (1) galvo, (2) piezo, and (3) stage scan modes. In the galvo scanner, the laser pulses move in the *xy* directions, whereas the photoresist remains stationary, and the *z-*drive movement is adjusted by either a stage motor or a piezo drive. In the piezo and stage scan modes, a piezo drive and a stage drive, respectively, move the stage in the *xyz* directions, whereas the laser beam remains fixed. The piezo scan mode may be used for the fabrication of high-resolution 3D structures, as it provides a large travel range in the *xyz* directions, whereas the galvo scan mode is better for the fabrication of larger structures, as the writing speed is faster. The stage scan mode can pattern significantly larger areas but with lower resolution than that of the other scanning modes. Through the integration of a complementary metal oxide semiconductor or charge-coupled device camera, real-time monitoring of the polymerization process can be achieved^67,72^.

In contrast to conventional microfabrication, no spin coating, no photoresist thickness control, and no soft baking or postexposure baking of the photoresist are required; the fabricated structure simply has to be developed after photoresist crosslinking. The photoresist is usually drop cast onto the substrate. Finally, the unexposed regions of negative-tone resist and laser-exposed regions of positive-tone resist are removed in a developer bath. Additional flood exposure by UV light may be performed after development to trigger additional chemical crosslinking of the photoresists.

## Microneedle array fabrication development

**Fig. 5:**
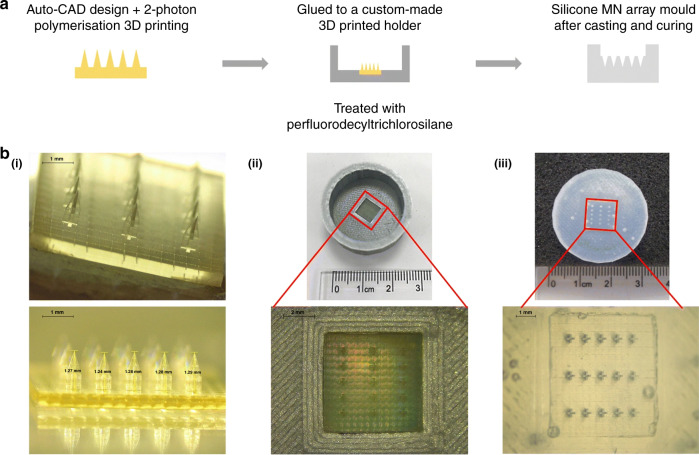
2PP and micromolding techniques to produce microneedle arrays. **a** Schematic image representing the steps for manufacturing the silicone microneedle array molds from the 2PP master microneedle arrays. **b** Light microscopy images of the fabrication process, showing the (i) master microneedle array fabricated by 2PP, (ii) master microneedle array placed on a PLA holder and a close-up image, and (iii) silicone negative mold of a microneedle array^72^. Reprinted from ref. ^72^ with permission.

2PP enables the fabrication of complex microneedle geometries due to its great flexibility and high resolution. In comparison to conventional microfabrication such as DRIE, no spin coating, no photoresist thickness control, and no soft-baking or postexposure baking of the photoresist are required; the fabricated structures need only to be developed after photoresist crosslinking. However, the quality of the fabrication significantly relies on the laser input, choice of material, and postexposure treatment. Microneedles manufactured via 2PP either are directly used for testing or have been used as master molds for the formation of the final microneedle arrays. In general, the micromolding process is divided into the following steps: (1) manufacturing of the master microneedle, (2) fabrication of microneedle array molds, e.g., in PDMS, and (3) fabrication of microneedle array replicas, which can be nondissolvable for biomedical testing or dissolving arrays for drug and vaccine delivery. Cordeiro et al.^73^ combined the 2PP and micromolding techniques to produce dissolving and hydrogel-forming microneedle arrays (Fig. 5). Microneedles are traditionally designed as solid or hollow microneedles. For solid microneedles, the therapeutics are coated on the microneedle surface and are dissolved after insertion into the skin; for hollow microneedles, the microneedle channel enables the transfer of fluid from and into the skin. However, almost any 3D microneedle structure can be fabricated with 2PP and different researchers have applied the technology to fabricate open-channel design microneedles^12^ or bioinspired microneedle geometries^16^.

**Fig. 6:**
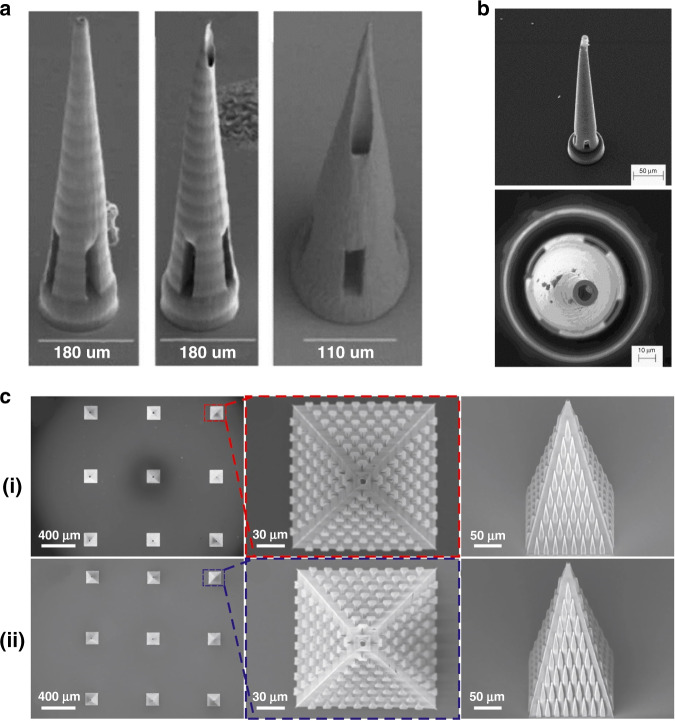
Microneedle array fabrication development by 2PP. **a** Microneedles fabricated from Ormocer® by 2PP with an 800 μm height and base diameters ranging from 150 to 300 μm^74^. Reproduced with permission from ref. ^74^. Copyright © 2007 John Wiley & Sons. **b** A microneedle made from Ormocer® with a 750 μm height and a 200 μm base diameter^12^. Reproduced with permission from ref. ^12^. Copyright © Elsevier. **c** Scanning electron micrograph (SEM) of a microneedle array (i) fabricated by 2PP from OrmoComp ® and 1 wt% Ciba® IRGACURE® 2959, including top and side views. SEM of epoxy replica (ii) microneedle array made from EPO-TEK® 353ND replicated from (i) through micromolding, including top and side views^15^. Reprinted from ref. ^15^ with permission.

Despite the high potential of 2PP technology, few studies to date have reported 2PP of microneedles for biomedical applications, including drug delivery and biosampling. For some specific applications, the microneedle geometries need to be more complex; in addition, sharper tips will facilitate penetration and reduce the microneedle insertion forces. To our knowledge, Doraiswamy et al.^13^ fabricated the first microneedles by 2PP from Ormocer^®^ (organically modified ceramics; Fraunhofer-Gescllschaft, Munich, Germany) hybrid materials with a 750 μm height and 200 μm base diameters^13^. Ormocer^®^ materials are amorphous organic–inorganic hybrid materials, which are formed by sol-gel procedures from liquid precursors. The materials contain organic monomers, organically modified silicon alkoxides, and metal alkoxides. During the 2PP process, powerful covalent bonds are created between the polymer and ceramic contents of the materials. Organic contents such as methacrylate groups are crosslinked by thermal, light, or redox-initiated processes, and inorganic contents such as alkoxysilane precursors crosslink and create inorganic Si-O-Si networks via controlled hydrolysis and condensation of organically modified silicon alkoxides^13,74^. Eventually, a 3D network is formed from the crosslinking of organic and inorganic contents of materials that inhibits separation of the material into isolated phases^13^. Microneedles made from Ormocer^®^ have been shown to remain intact after insertion into porcine skin^13,75^. The same group later fabricated more complicated microneedle geometries with 2PP in a single-step process, which was not possible with other established methods. In this study, arrays of microneedles with flow channels positioned at the center and off-center with respect to the needle tip with an 800 μm height and base diameters ranging from 150 to 300 μm were fabricated from Ormocer^®^ US-S4 (Fig. 6a)^75^. In these studies, despite fabricating microneedle geometries, which require multistep processes through conventional manufacturing methods, only low resolution and tip sharpness were achieved. Figure 6b shows the manufacturing weaknesses of the microneedle tip, which could exist due to incomplete polymerization of the material. Figure 6c represents an example of a more controlled 2PP fabrication for producing microneedle arrays developed by other researchers^16^. However, the microneedle arrays shown in Fig. 6a, b successfully penetrated cadaver porcine adipose tissue without fracture. Later, the same group used 2PP and micromolding processes with PDMS to produce round-tip polymeric microneedles 500 μm in height and 150 μm in base diameter from acrylate-based polymers. The study showed that microneedle arrays can withstand a 10 N axial load and successfully penetrate the human SC and epidermis without fracture^14^. In another study, a combination of 2PP and micromolding was used to make Ormocer^®^ microneedle replicas; to provide antimicrobial functionality on the surface, pulsed laser deposition was used to deposit silver thin films^76^. 2PP is usually combined with other techniques such as soft embossing or micromolding to create polymeric microneedles. This provides the benefits of high-fidelity replication molding with the precision, accuracy, and design freedom of 2PP. In 2010, Doraiswamy et al.^68^ manufactured hollow microneedle arrays in a single-step process with diverse aspect ratios from Ormocer^®^ materials and showed their suitability for the transdermal administration of a PEG-amine quantum dot solution. In this study, the microneedles fabricated from OrmoComp^®^ were able to penetrate the SC of pig skin and, consequently, were able to distribute the quantum dots in the epidermis and dermis^68^. The ultimate application in this case is encrypted identification.

In more recent years, there has been high interest in utilizing commercial 2PP systems for the fabrication of microneedle arrays. Table 2 provides a detailed review of the research works using 2PP for fabricating microneedle arrays. For example, Liao et al.^20^ fabricated microneedle structures for pharmaceutical delivery using a commercial photonics professional system (Nanoscribe GmbH, Germany) with the IP-S photoresist having a high elastic modulus *E*_Young_ ≈ 2–3 GPa. The fabricated structure penetrated the skin without fracture^20^. In another example, Cordeiro et al.^73^ fabricated a variety of microneedle arrays between 900 and 1300 µm in height, 300 and 500 µm in base width, and 100 and 500 µm in interspacing, using a commercially available system with relatively complex conical, cross-shaped, pyramidal, and pedestal shapes. Microneedle arrays were created to produce negative silicone molds and consequently exploited to create dissolving and hydrogel-forming microneedle patches. The fabricated microneedle arrays were inserted into skin models consisting of stacked layers of Parafilm and porcine skin to evaluate the insertion properties and drug delivery efficiency of different designs. The study demonstrated arrays with pyramidal and conical needle profiles exhibiting the greatest depth of insertion (64–90% of the total microneedle height) and greater rate of drug delivery after ex vivo and in vitro applications^73^. Mechanical stability of the patches is a key factor in successful insertion and finally drug delivery. Microneedle arrays without sufficient mechanical strength and stiffness will experience damage when entering the skin. Researchers have used the technology for drug delivery into difficult-to-access locations of the body. For example, Aksit et al. used 2PP and electrochemical deposition to produce gold-coated copper microneedles for drug delivery into the inner ear of a guinea pig in vivo. In this study, 2PP was used to directly print the negative mold of the 3D microneedle structure. Subsequently, electrodeposition of copper was applied to the negative mold to create copper microneedles and the 3D microstructure obtained was coated with a layer of gold by the immersion deposition method to improve the surface biocompatibility. The microneedle template was successfully penetrated into the round window membrane (RWM) of the middle ear of a guinea pig. The final microneedle had a tip radius of curvature of 1.5 µm, height of 430 µm, shaft diameter of 100 µm, and base diameter of 405 µm^77^. The same group also used 2PP to make microneedles for drug delivery into the guinea pig inner ear through microperforation of the RWM^78,79^ and into a human RWM^80^.

**Fig. 7:**
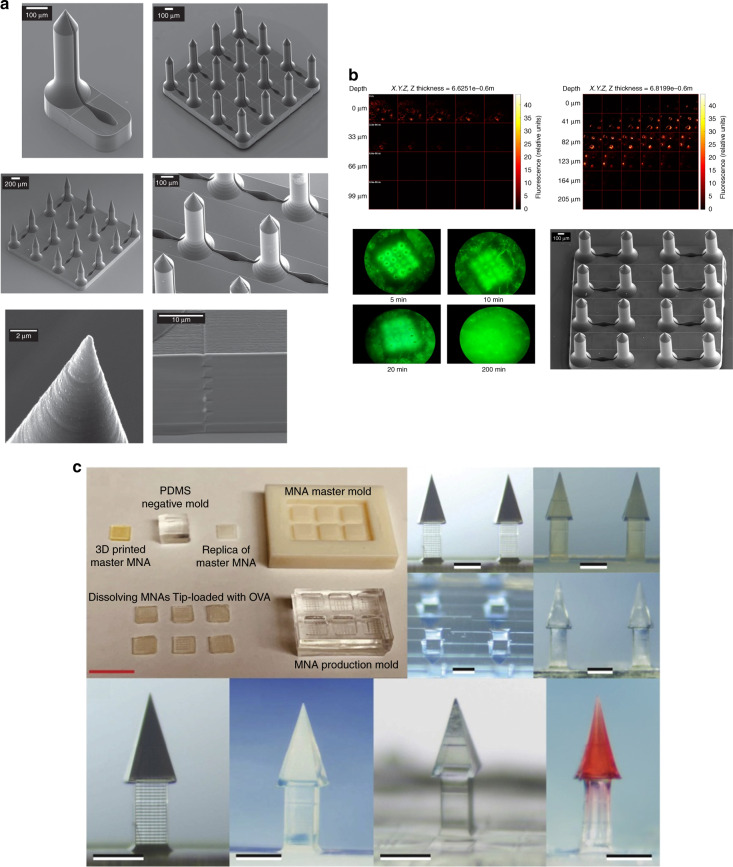
Microneedle array fabrication development by 2PP. **a** SEM of multiple designs of a single microneedle and arrays of microneedles with open-channel designs connected to microfluidic reservoirs. Microneedles have a 700 μm height and a flange design at the base with a 150 μm height. **b** Multiphoton microscopy image representing the diffusion of fluorescein solution underneath the skin surface of a rabbit ear (top-right image) and the topical application of solution on the tissue surface as a control (top-left image) after insertion of an array of 16 microneedles^11^. Reprinted from ref. ^11^ with permission. **c** Manufacturing steps for producing dissolving microneedles loaded with different drugs, including cabotegravir sodium and ibuprofen sodium^17^. Reprinted from ref. ^17^ with permission.

2PP enables the reproducible production of complex microneedles in a relatively simple and reliable manner. For example, Faraji Rad et al.^12^ fabricated microneedles with open microfluidic channels via 2PP and microneedle arrays successfully penetrated rabbit ears without fracture (Fig. 7a, b). In another study, 2PP and micromolding were applied to create dissolving microneedle arrays with complex undercut geometries; the tips of the microneedles were loaded with a model antigen (ovalbumin) with an adjuvant vaccine component (poly(I:C)). The arrays were able to penetrate human and murine skin for cutaneous vaccination^18^ (Fig. 7c).

Although Nanoscribe GmbH is currently dominating the market, researchers have demonstrated the use of other commercially available systems. Plamadeala et al.^16^ used the Workshop of Photonics^®^ to produce microneedle arrays with pyramidal shapes with a height of 210 µm, a square base of 160 µm, and a tip sharpness of ~1 μm. The microneedle design is bioinspired by a bug structure, which allows passive transportation of liquid on the lateral surface of the microneedles. A laser beam of wavelength 515 nm with a 290 fs pulse duration and a 0.42 NA objective lens with writing speeds of 3 and 1 mm s^−1^, and power levels of 2.4 and 1 mW were used to fabricate the microneedles. These studies demonstrate that 2PP facilitates the fabrication of more complex shapes of microneedles in a fast and reproducible manner, which would enable the application of microneedle arrays for more critical clinical applications. A synergistic combination of 2PP and micromolding methods enables an effective and easy high-fidelity replication of microneedle arrays^12^. 2PP enables the direct writing of microneedles without the need for dry or wet etching processes for tip formation. This allows the microfabrication of larger areas and microneedle heights not attainable by any other micro/nanomanufacturing method. However, the high operation cost and writing speed of 2PP are limiting factors for the commercial development of microneedle patch array products, which require high-volume, low-cost manufacturing methods. Therefore, its main role will be as a prototyping tooling method rather than as a large-scale manufacturing technology. This problem can be addressed by the development of rapid replication molding methods using 3D laser stereolithographic tools, currently in the prototype stage, or by reel-to-reel manufacturing.

## Conclusions

Extensive research has been carried out on the design, fabrication, and application of microneedle systems. Microneedles will be welcomed from both patients and the public health perspective due to the increase in comfort and convenience of application through point-of-care applications. The choice of manufacturing technique for the production of microneedles is dependent on the material properties, fabrication cost, and desired length and shape of the microstructure. Despite decades of research and the superior advantages of microneedles in application, the number of licensed microneedle patch devices that have entered the medical device industry is limited. Most research is at the proof-of-concept phase rather than exploring the clinical phase of the technology. This is partly because of various manufacturing and technical issues associated with microneedle array production. Although many studies have claimed robust and cost-effective production of microneedles, to date, none of the fabrication methods have reached the medical industry. Undoubtedly, the most challenging problem has been the development of low-cost manufacturing methods that will enable the clinical implementation of this technology. Recent advances in emerging technologies such as 3D laser lithography systems show promise, eliminating previous drawbacks related to the design and fabrication of microneedle-based devices so that more clinical aspects can be investigated in the near future.

As new fabrication technologies are emerging, there is increasing scope for reducing both the cost and time required to manufacture microneedle devices. Additive manufacturing techniques in particular have emerged as a promising method for the fabrication of 3D micro/nanostructures, and in recent years, the 2PP technology has shown great flexibility and higher resolution in comparison to earlier microneedle fabrication techniques. 2PP enables the fabrication of complex functional components in a single step without the need for integration or the assembly of parts. Thus, feature geometries and resolution are no longer limited by the physics of etching or machining but are precisely rendered from CAD data. 2PP enables the direct writing of microneedles without the need for dry or wet etching processes for tip formation or the integration of parts and allows the microfabrication of larger areas and microneedle heights not attainable by any other micro/nanomanufacturing method. This method enables the creation of any kind of 3D structure with the possibility to integrate parts into a completed device in a single-step process with sub-100 nm resolution^12,81^, unlike conventional 3D printing, which does not have the resolution required for microneedle arrays. However, the process is limited to the use of photocrosslinkable materials only and has a slow processing speed if fabricating large microstructures or printing at superior resolution^82^. To date, the 2PP writing speed has been a limiting factor for the commercial development of microneedle patch array products that require high-volume, low-cost manufacturing methods, and 2PP is currently viewed mainly as a prototyping tooling method rather than a large-scale manufacturing technology. This may well change in the future through the development of rapid replication molding methods using 2PP prototypes, enabling inexpensive, mass-produced microneedle patches to be used clinically at the point of care for theranostics and vaccination.

## Supplementary information

Permission for reprinting image

Permission for reprinting image

Permission for reprinting image

Permission for reprinting image

Permission for reprinting image
